# Social science for climate-resilient grasslands: Strategies to support individual and systemic adaptation

**DOI:** 10.1016/j.isci.2026.116265

**Published:** 2026-06-08

**Authors:** Sarah Gonzalez-Coffin, Christine D. Miller Hesed, Heather M. Yocum, Ryan Roberts

**Affiliations:** 1 Department of Psychology and Neuroscience, University of Colorado Boulder, Boulder, CO 80303, USA; 2 Cooperative Institute for Research in Environmental Sciences, University of Colorado Boulder, Boulder, CO 80303, USA; 3 North Central Climate Adaptation Science Center, University of Colorado Boulder, Boulder, CO 80303, USA; 4 Playa Lakes Joint Venture, 700 Ken Pratt Blvd, Suite 206, PMB 338, Longmont, CO 80501, USA

**Keywords:** health sciences, environmental science, social sciences

## Abstract

Climate change interacts with existing stressors and contributes to the degradation and transformation of grassland ecosystems in the Great Plains of the United States, posing challenges for human communities in the region. Understanding the human dimensions of grassland systems is essential for developing adaptation responses that sustain grassland ecosystems and rural livelihoods. To address this need, we conducted a narrative-style review of social science research in the Great Plains, including anthropology, economics, sociology, political science, psychology, and communication studies. We present a novel application of a behavioral economics framework to grassland management, identifying nine social-science-backed strategies that conservation professionals can apply to advance climate adaptation through individual (*i-frame*) and systemic (*s-frame*) levels. We also highlight the need for a third, group-level (*g-frame*) dimension to be further theorized to present opportunities for future research to generate strategies that will address the needs of all grassland stewards.

## Graphical abstract

**Figure:**
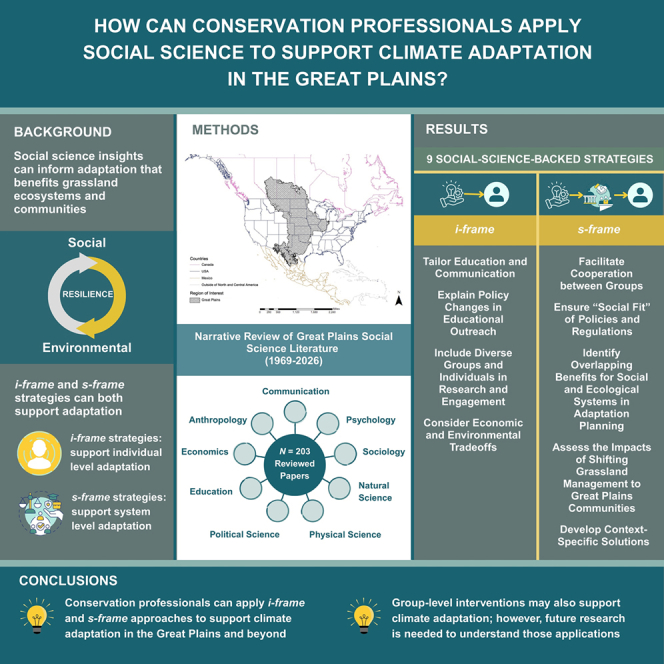

## Introduction

Effective adaptation to climate change requires the integration of physical, natural, and social sciences.^1^ While past approaches to environmental problems have emphasized the ecological aspects of solutions, a growing consensus recognizes that humans are deeply intertwined with the environment, contributing to both conservation and degradation.^2^^,^^3^^,^^4^^,^^5^ Environmental management decisions and practices, both individually and collectively, can either bolster or dampen the long-term resilience of ecological systems to climate change.

Grassland management in the Great Plains of the United States provides an excellent example of how social and ecological systems are intimately connected through complex and dynamic interactions and feedback. While considerable headway has been made in understanding ecological aspects of grassland systems in the Great Plains, the understanding of the human dimensions remains inadequate for supporting adaptation to climate change.^6^ Recent studies have made important contributions to addressing this gap, for example, by synthesizing grassland managers’ perceptions and attitudes toward prescribed burning,^7^ and reviewing the state of rangeland social science since the 1970s to identify research gaps.^8^ However, there has yet been no effort to review the Great Plains social science research to synthesize applications for supporting adaptation to climate change. We address this gap by conducting a narrative review of the Great Plains social science literature to identify strategies for *conservation professionals*—those who design, fund, and implement natural resource management plans and policies in collaboration with private landowners and caretakers—to support climate adaptation in the Great Plains. Definitions for all terminology used in this paper are provided in Table S1.

This review is both more expansive and more applied than previous reviews of Great Plains social science.^7^^,^^8^ First, the review is grounded in broadly shared grassland management questions that, if answered, would help to support collective conservation of the grasslands in the northern Great Plains of the United States in a changing climate^6^^,^^9^^,^^10^ and complements a recent synthesis of climate and ecological science to address those questions.^9^ Second, this review uses a novel application of the *i-frame* and *s-frame* behavioral economics framework^11^ to identify social-science-backed strategies to support climate adaptation in grassland management at the individual and systemic levels. Thus, our review is among the first to translate social science literature into actionable strategies that conservation professionals can use to support climate adaptation across the Great Plains. While the analysis we present is focused on the Great Plains of the United States, we expect that many of the strategies identified could ultimately be applied to support adaptation in grassland ecosystems around the world.

### The Great Plains

**Figure 1:**
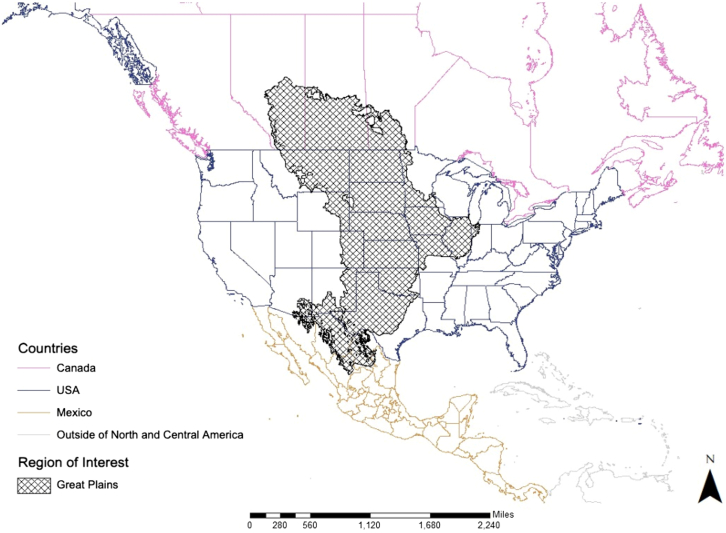
Modified map shows the Great Plains region in North America

The Great Plains (Figure 1) is one of the most economically and ecologically important regions in the United States.^12^^,^^13^ The grassland ecosystems of the Great Plains have been in a dynamic relationship with people since time immemorial, and Indigenous peoples living in the Great Plains helped to shape a highly heterogeneous and biodiverse grassland landscape by setting fires and hunting.^14^^,^^15^ Beginning in the 1850s, European settlers colonized the Great Plains and initiated the rapid conversion of grasslands to cropland.^16^ Today, approximately 83% of the North Central Great Plains is privately owned and managed.^6^^,^^17^ This means that grassland conservation and restoration must be a collaborative effort that includes private landowners and caretakers—hereafter referred to as *land stewards*—alongside federal, state, and Tribal agencies and non-governmental organizations.

Human communities are inextricably linked with the grassland ecosystems in which they reside, relying on them to provide many resources and amenities, including food, energy, and recreational opportunities. A precipitation gradient that decreases from east to west influences the character of grassland ecosystems, with tallgrass in the east transitioning to shortgrass in the west. The same precipitation gradient has shaped agriculture and land use across the region, with rain-fed, row-crop agriculture dominating the eastern portion, and irrigated agriculture or ranching dominating the western portion.^18^ Agriculture and energy development (both fossil fuels and renewable energy) are economically important to communities on the Great Plains and provide food and fuel domestically and abroad,^19^ yet these activities are also among the primary drivers of grassland loss.

The impacts of climate change—including increased temperatures, variability in precipitation, and concentration of carbon dioxide in the atmosphere—affect the grassland ecosystems and human communities of the Great Plains in many complex and interacting ways.^18^^,^^19^ Extreme weather events and interannual variability influence water availability across the region, exacerbating hazards such as floods and drought. Rising temperatures contribute to increased risk and severity for wildfires, while changes in vegetation composition and diversity shape the quality and availability of food sources, as well as habitats for thousands of grassland species. Synergistic interactions between climate change, environmental disturbances, and existing stresses from socioeconomic processes such as demographic shifts have significant implications for the health of grassland ecosystems and the well-being of rural communities. Conservation professionals must contend with these changes and support land stewards as they work to conserve grasslands and sustain the human, animal, and plant communities that depend on these landscapes.^20^

### i-frame and s-frame strategies to supporting climate adaptation in the Great Plains

Successfully responding to the impacts of climate change requires adaptation at both the individual and systemic levels to enable climate-resilient management.^21^ In this paper, we use *climate-resilient* to refer to the capacity of grassland ecosystems and human communities living in those grasslands to adapt to climate change. Behavioral economists describe approaching problems at the individual level or the systemic level as the *i-frame* or *s-frame*, respectively. Supporting climate-resilient management of grasslands at the individual level, or *i-frame,* emphasizes changing individual attitudes, decisions, and behaviors and is predominantly studied in the fields of behavioral science and psychology. For example, education campaigns that provide information about climate science and encourage positive attitudes toward conservation can motivate individual farmers to install buffer strips or two-stage ditches to reduce erosion and runoff of fertilizers and pesticides.^22^ Supporting climate-resilient management at the systemic level, or *s-frame*, focuses on changing the systemic conditions that constrain and enable individual behaviors, and is generally studied by the fields of policy, sociology, economics, and law.^11^ For example, programs for cost-sharing and technical support, such as the U.S. Department of Agriculture Environmental Quality Incentives Program (EQIP), lower the financial barriers for farmers to plant cover crops, an important climate adaptation in increasingly drought-prone areas in the Great Plains.^23^

The *i-frame* and *s-frame* behavioral economics framework is useful for distinguishing between strategies that target individual change and those that address systemic constraints. This distinction is important for considering what type of strategy is most needed to support climate-resilient grassland management. For example, a farmer may have positive attitudes toward modifying grazing practices during drought to reduce soil degradation and preserve forage, but be constrained by grazing permit schedules that limit flexibility. Developing an educational campaign to change the farmer’s attitude (an *i-frame* approach) would not be beneficial in this instance when what is needed is a revision of grazing permit regulations (an *s-frame* approach). Thus, the *i-frame* and *s-frame* provide an intervention-relevant structure to identify appropriate individual and systemic adaptation strategies for conservation professionals and policymakers.

Other frameworks for understanding and managing complex social-ecological systems exist, but are not as well-suited to identifying ways to support adaptation at the individual or systemic level. For example, the social ecological systems (SES) framework^4^^,^^5^ offers an analytical approach to identify and describe systems for making decisions about managing the environment and using natural resources across multiple spatial and time scales. The SES framework has been successfully used to describe the context in which natural resource management decisions play out and the impacts of those decisions, but it is less useful for identifying a range of potential strategies for supporting adaptation to climate change. Resist-accept-direct (RAD) is another framework that supports climate adaptation by helping natural resource managers decide when to adjust management strategies in the face of ecological changes.^24^^,^^25^^,^^26^ While the RAD framework informs a particular management strategy in a well-defined situation, it is not as useful for analyzing or organizing a general set of strategies to support climate adaptation across the Great Plains, where grassland managers have differing goals, challenges, and opportunities.^10^ Furthermore, a recent review of social science research on agricultural practices emphasized the need for more systems-level interventions and less focus on individual-level strategies.^27^ Therefore, we selected the *i-frame* and *s-frame* as our analytic and organizational structure to identify strategies for grassland managers and policymakers to advance climate adaptation.

## Methods

### Scoping the study

**Figure 2:**
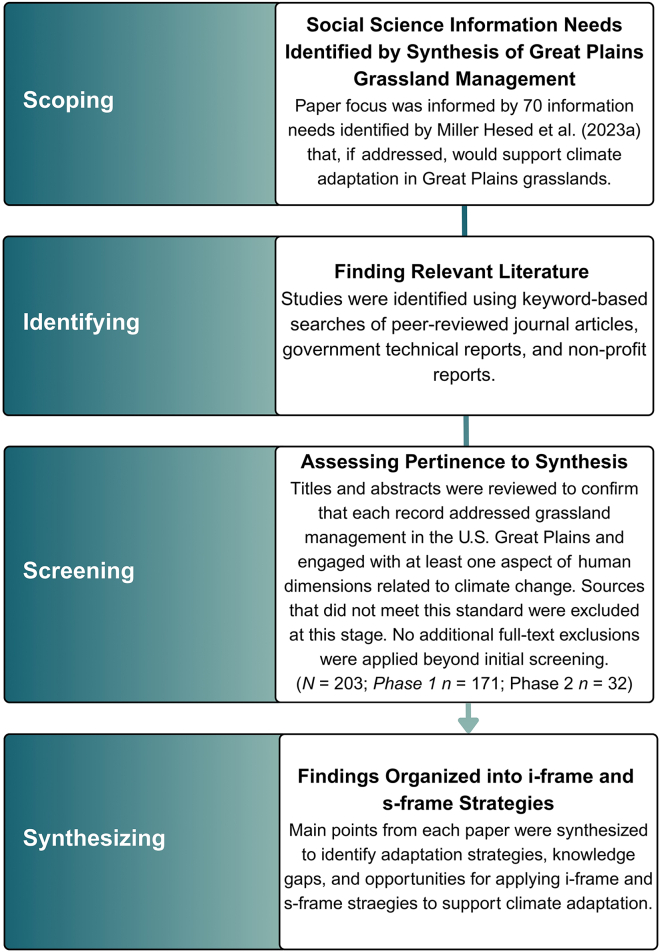
Narrative review and synthesis process

The objective of our study was to identify how existing social science in the Great Plains can inform conservation professionals, including land managers and policymakers, in addressing grassland adaptation in a changing climate. Since grassland management goals, challenges, climate vulnerabilities, and management practices in the Great Plains are so varied,^6^^,^^10^^,^^20^ a standard systematic review of the literature was not a feasible approach for meeting our objective. To capture the breadth of social science relevant to climate-resilient grassland management in the Great Plains, we instead conducted a narrative literature review—an exploratory approach that facilitates synthesis across disciplines and perspectives^28^^,^^29^^,^^30^ (Figure 2). Unlike systematic or scoping reviews that focus on extensively cataloging studies based on predefined criteria, a narrative review approach allowed us to broadly synthesize social science that varied widely in discipline, topic area, methodology, and geography.^31^ This approach also paralleled a recent synthesis of the physical and natural science literature to support the adaptation of grassland ecosystems in the Great Plains, positioning this manuscript as a complementary review of social science that responds to questions identified in that work.^6^^,^^9^^,^^10^ We followed the Scale for the Assessment of Narrative Review Articles (SANRA) guidelines for a high-quality narrative review.^32^

### Identifying relevant literature

We identified relevant literature in two phases. The first phase was conducted between May and August 2023 and identified 171 sources published between the years of 1969–2023. The second phase was completed in February 2026 and used the same search criteria, but included only papers published from September 2023 through February 2026. This second phase of literature identification was conducted to incorporate the most recently published social science and resulted in the identification of 32 additional papers for screening. In both phases of literature identification, we used the same search strategy. We searched for articles in Google Scholar using terms for grassland management and responses to climate change (“Adaptation,” “Climate change,” “Manag∗,” “Mitigation”), region (“Great Plains,” “Prairie,” “Central Grasslands,” “Central Grassland Flyway,” “Colorado,” “Kansas,” “Montana,” “Nebraska,” “New Mexico,” “North Dakota,” “Oklahoma,” “South Dakota,” “Texas,” and “Wyoming”), stakeholders and rightsholders (“Communit∗,” “Corporations,” “Ejidos,” “Farmers,” “Federal Resource Managers,” “General public,” “Government,” “Grassland Communities,” “Indigenous Nations,” “Landowners,” “Land Managers,” “Non-governmental,” “Pastoralist,” and “State Resource Managers”), and social science (“Social Science”). Examples of search strings we used include combinations of the keywords above, including “social science” AND “adaptation” and “grassland communities”’ AND “climate change.”

### Screening identified literature

We screened papers identified in both phases of literature identification through a keyword search to ensure their relevance to human dimensions of grassland management and/or climate adaptation in the Great Plains. We excluded sources that did not include one or more of the key search terms or that did not include any social science. We also cross-referenced articles with the fifteen main categories of social science information needs identified by the complementary ecological synthesis,^6^ prioritizing the inclusion of studies that conveyed adaptation strategies applicable to grassland management. We included peer-reviewed journal articles, government technical reports, and nonprofit publications. Our final collection of literature for review and synthesis (available at https://doi.org/10.17632/sv5wncbktc.1) included 203 sources spanning the fields of psychology (*n* = 97), economics (*n* = 88), political science (*n* = 65), sociology (*n* = 77), communication (*n* = 51), education (*n* = 41), and anthropology (*n* = 31). Many papers were interdisciplinary, integrating social sciences with natural (*n* = 99) and/or physical sciences (*n* = 27).

### Synthesizing literature to identify themes

The body of literature we synthesized pertained to numerous stakeholder and rightsholder groups, including pastoralists/ranchers (*n* = 67), landowners (*n* = 51), farmers (*n* = 40), state resource managers (*n* = 32), federal resource managers (*n* = 29), the general public (*n* = 23), non-government organization staff (*n* = 20), Indigenous/Tribal members (*n* = 6), corporations (*n* = 5), and other groups (academic/experts, conservation advocates, politicians/judges, and so forth) (*n* = 41). We reviewed this body of literature with a specific focus on identifying and synthesizing strategies relevant to the needs identified in the review by Miller Hesed and colleagues.^9^ However, the broadly shared grassland management information needs identified in that synthesis were not evenly addressed in the social science literature that we reviewed (Tables S2 and S3), with the greatest number of articles and reports addressing how conservation can be achieved on private grasslands (*n* = 78) and best practices for grassland restoration in a changing climate (*n* = 68). In contrast, very few articles and reports addressed human dimensions relevant to the questions of how climate change will impact herbaceous invasives (*n* = 6), grazing practices (*n* = 11), and conservation of animal species of concern (*n* = 7).

As we reviewed the literature, themes relevant to supporting climate adaptation in the Great Plains emerged. We organized these themes into two interconnected levels: *i-frame* strategies that support adaptation at the individual level and *s-frame* strategies that support adaptation at the systemic level. Specifically, we identified in the social science literature the individual-level human dimensions that can inform how conservation professionals provide technical support, services, and advice to land stewards. We also identified systemic-level insights that will be more relevant to researchers, policymakers, and adaptation planners.

## Results

The results presented here are written primarily for two audiences: conservation professionals and researchers who support land stewards. We draw upon our review and synthesis of the social science literature to describe *i-frame* and *s-frame* strategies that can support the adoption of adaptation practices to enhance grassland resilience to climate change, facilitate sustainable agricultural productivity, and reduce greenhouse gas emissions (Table 1). We also list quantitative indicators for each of the nine social-science-backed strategies that could be used to evaluate the effectiveness of these practices in building grassland resilience to climate change; however, further research is needed to develop and refine these quantitative metrics.

**Table 1:** i-frame and s-frame strategies to support grassland adaptation to climate change, relevant studies from our review, and examples of strategy application in a grassland context

Strategy	Relevant Studies	Examples of How to Apply the Strategy in Grasslands
***i-frame***		

Tailor education and communication	Morton et al.,^33^ Sanderson et al.,^34^ Wilmer et al.,^35^ Wilmer.&Sturrock^36^	focus educational communications on the effectiveness of prescribed burning for controlling eastern redcedar (considered a problematic species by most landowners in the region) to encourage greater uptake of the practice^33^
Explain the implications of policy changes in educational outreach	Hinojosa et al.,^37^ Kreuter et al.,^38^ Roberts et al.^39^	provide information about how negligence standards have changed for prescribed burning when engaging in outreach with landowners^40^
Include diverse groups and individuals in research and engagement	Bruno et al.,^8^ Booth,^41^ Brunson et al.,^42^ Jamar et al.^43^	use methods such as stakeholder mapping to gain a more comprehensive understanding of the diversity of grassland stakeholders and their varied interests and needs^8^
Consider economic and environmental trade-offs	Carrasco Galvan et al.,^16^ Kreuter et al.,^38^ Shogren et al.^44^	provide financial incentives for landowners to participate in grassland or wildlife conservation programs^45^

***s-frame***		

Facilitate cooperation between groups	Cole et al.,^46^ Cole et al.,^47^ Hultquist et al.,^48^ Romsdahl et al.,^49^ Wardropper et al.,^50^ Wiest et al.^51^	create spaces for different groups to meet and discuss their various grassland management objectives and motivations^50^
Ensure “social fit” of grassland conservation practices and regulations	Ayambire et al.,^52^ Carolan,^53^ Davidson et al.,^54^ Gosnell et al.,^55^ Pape et al.^56^	design conservation policies to meet local needs and values, such as conserving at-risk grassland species while supporting rangeland operations that drive the local economy^52^
Identify overlapping benefits for social and ecological systems in adaptation planning	Ahlering et al.,^57^ Bentley Brymer et al.,^58^ Leis et al.^59^	restore damaged grassland ecosystems to bolster ecological resilience while also reducing regional public health issues, such as allergies from pollen^56^
Assess the impacts of shifting grassland management to Great Plains communities	Sanderson et al.,^34^ Gosnell et al.,^60^ Haggerty et al.,^61^ Haggerty et al.,^62^ Emmet Jones et al.,^63^ Joshi et al.,^64^ Mendham et al.^65^	track changes in residency across the Great Plains over time to quantify the magnitude of amenity migration in rural areas and identify changing values and land use^60^
Develop context-specific solutions	Wilmer & Sturrock,^36^ Enck & Cojocariu,^66^ Thiault et al.^67^	use Public Participatory GIS (PPGIS) to understand local social and economic dimensions relevant to climate-resilient grassland management^66^

*i-frame* strategies target individual human dimensions such as values, beliefs, and attitudes. *s-frame* strategies target the systems that constrain or enable the ability of individuals to adopt adaptation practices.

### i-frame strategies for supporting adaptation practices

Individual differences can present challenges for coordinating grassland management strategies across the region, especially given the divergent and sometimes conflicting interests of land steward groups comprising the grassland landscape. Yet these differences among land stewards also offer opportunities for applying different *i-frame* strategies to support adaptation and mitigation actions at the individual level in response to climate change. Differences in attitudes, values, motivations, ideologies, and demographics—such as age, gender, income, location, and education—shape how managers make decisions.^34^^,^^68^^,^^69^^,^^70^^,^^71^^,^^72^ Personal experience, perceived control, and uncertainty further influence behavior.^64^^,^^73^ Risk perceptions drive the choice between familiar practices and novel but uncertain options, with many managers avoiding innovations unless potential losses are clear and immediate.^70^^,^^74^ Political ideology affects which climate signals managers notice, the concern they express, and their sense of efficacy; liberals report greater perceived risk and agency to address climate change than conservatives.^35^^,^^75^^,^^76^^,^^77^^,^^78^ Collective norms, interpersonal networks, and a ranching culture grounded in self-reliance and neighbor support can either spread or suppress new practices.^68^^,^^79^^,^^80^^,^^81^^,^^82^ Demographic shifts, land-use change, and rural restructuring are reshaping these social landscapes, creating new constraints and openings for management interventions.^59^^,^^60^^,^^83^^,^^84^ Recognizing and mapping this heterogeneity allows extension programs and policy designers to tailor messages, incentives, and decision aids to specific segments of the population, transforming variability from an obstacle into a tool for accelerating climate-resilient management.

Based on our review, we identified four *i-frame* strategies for supporting climate adaptation practices in the Great Plains grasslands: 1) tailoring education and communication, 2) explaining the implications of policy changes in outreach, 3) including diverse groups and individuals in research and engagement, and 4) considering economic and environmental trade-offs. By focusing on these opportunities, social science insights can be applied to address barriers to resilient social and ecological systems in grassland regions.

### Tailor education and communication

Tailored education and communication can enable climate-resilient grassland management by making adaptation practices more personally and contextually relevant to land stewards, while achieving community outreach goals of providing information that supports decision-making. Deciding to adopt—or scale up existing adaptation practices—requires grassland managers to participate in information sharing among trusted peers (e.g., neighbors and friends) or federal education programs (e.g., U.S. Fish & Wildlife Climate Change and Adaptation Training workshop). Climate change impacts vary across regions, and risks and benefits of adaptation are not uniformly experienced, creating a need for grassland managers to filter which adaptation methods are most relevant and cost-effective.^75^ Rural communities are not a monolith and vary widely in livelihoods, values, and cultural context.^85^ Rather than viewing heterogeneity as a barrier to educational campaigns or participation in programs, conservation professionals can segment messaging about adaptation practices to smaller audiences, addressing the specific needs, values, and lived experiences of individual land stewards.^84^

Tailored information campaigns have been used to equip ranchers with rotational grazing strategies to promote soil health and carbon storage,^86^ provide satellite-based RangeSAT forage alerts that signal biomass thresholds to avoid overgrazing and protect vegetation cover,^87^ and issue drought bulletins that forecast water scarcity and prompt pre-emptive herd reductions to preserve resources.^86^ In another example, drought-prone areas where fires and water shortages are prevalent have facilitated the adoption of prescribed burning to manage fire risks when directly tailoring communications to address perceived risks about legal liability associated with burning.^64^^,^^70^ Alleviating risk concerns allows grassland managers to weigh the appropriateness of prescribed burning as a method in their adaptation toolkit. Quantitative indicators that can be used pre- and post-campaign to measure the impact of tailored education and communication outreach efforts on different audience segments. This may include changes in audience attitudes and behavior that can be measured, such as differences in perceived risks of adopting new practices (e.g., 1–5 Likert scale of agreement), rates of practice adoption (e.g., percent of ranchers using rotational grazing), or changes in perceived climate-related risks (e.g., drought severity).

Economic concerns and attachment to familiar management practices can also shape receptiveness to adopting new practices. As climate-related uncertainty increases, land stewards may weigh the costs, labor, and compatibility of new strategies against the stability of existing operations.^88^^,^^89^^,^^90^ Tailoring communication to address these concerns by clarifying potential trade-offs and emphasizing continuity with local land ethics can foster informed decision-making. In the Grand River Grasslands of northern Missouri and southern Iowa, for instance, only half of the landowners’ view prescribed burning as a legitimate management tool.^33^ However, outreach that framed fire as a method to control Eastern Redcedar, a species many landowners consider problematic, can be more persuasive.^33^ Emphasizing operational and ecological benefits allows land stewards to assess adaptation tradeoffs through the lens of their livelihoods. Outreach that frames climate impacts such as drought, wildfire risk, or declining forage quality as shared regional challenges can build a sense of collective responsibility and cooperation locally.^58^^,^^59^ Appeals to local stewardship values, such as protecting water resources for future generations of farmers and ranchers, can encourage uptake of adaptation practices.^34^^,^^35^^,^^36^^,^^91^ In contexts where climate skepticism remains high, communicators can emphasize observable trends, such as shifting rainfall patterns or increased woody encroachment, which can foster dialogue and engagement around shared management goals, rather than using abstract climate terminology.^92^

### Explain the implications of policy changes in educational outreach

Educational outreach plays a critical role in helping managers navigate new rules, incentives, and resources. Policy changes that support climate-resilient grassland management are only effective if land stewards understand how those changes apply to them. State and federal resource managers, non-profit staff, and educators can support land steward uptake of adaptation practices by communicating about policies and their evolution in ways that reduce uncertainty and support informed, individual decision-making. For example, federal legislation such as the Inflation Reduction Act of 2022 expanded investments in climate adaptation and mitigation through funding for conservation programs, clean energy technologies, and agricultural incentives. More recently, the “Unleashing American Energy” executive order (Exec. Order No. 14154, 2025) redirected and reduced investments, shifting the availability of funding. Information about such investments is insufficient in motivating behavior^93^; clear communication about how these shifts in the status quo affect eligibility for cost-share programs, participation in conservation initiatives, or access to technical assistance is essential. Explaining these implications through trusted local channels, such as conservation districts, cooperative extension programs, or producer networks, can help land stewards translate national policy changes into concrete management decisions.

Addressing how policy changes may affect landowners’ risk is particularly important in educational outreach campaigns. For instance, legal liability has historically deterred the use of prescribed burning due to concerns about the costs of fire damage.^38^ Gross negligence standards offer some legal protection and are generally more appealing to landowners than simple negligence standards, which offer little to no protection regardless of fault.^39^ Educational outreach that clearly explains these legal shifts, including changes to statutes and liability protections, can help reduce perceived risks among land stewards.^93^ Doing so may increase landowner confidence and expand the use of prescribed fire and other adaptation practices.^37^^,^^38^ Quantitative indicators that can measure the effectiveness of educational outreach efforts can include measuring baseline public understanding of policies through online, phone, or mail-based survey measures, for example, as well as measuring changes in understanding over time (e.g., self-reported understanding on a 1–7 Likert scale).

### Include diverse groups and individuals in research and engagement

Efforts to support and inform climate-resilient management in the Great Plains must include the diverse perspectives, knowledge sources, experiences, and values of land stewards. By ensuring that diverse groups and individuals are meaningfully included, researchers, federal and state resource managers, and non-government organization staff can support the development and use of adaptation practices that reflect and respond to the varied needs of stakeholder and rightsholder groups across the Great Plains.

Our review identified a need for broader representation of stewards in peer-reviewed social science literature on grasslands—particularly those who identify as Black, Hispanic/Chicano, and Native American or Indigenous. These communities hold valuable knowledge and experience in regionally tailored grassland management,^94^ such as reintroducing bison herds on Tribal land.^41^ Despite increasing demographic diversity in the Great Plains,^42^^,^^95^ social science research about adaptation is not representative across stakeholders with different gender, ethnic, or socioeconomic identities.^8^^,^^43^ Further, we found that many social science studies in the region did not report participant demographics or examine how demographic variation influences adaptation outcomes, a pattern consistent with prior reviews.^8^ This oversight limits understanding of the social and cultural processes, information sharing, and decision-making dynamics that are involved in adapting to climate change by updating current grassland management practices.

Just as conservation professionals and land stewards monitor ecological and climate conditions to guide adaptive practices, research and engagement efforts should track social change and assess whether current management strategies meet the evolving needs of different communities. Longitudinal and multi-regional studies, such as those used by the U.S. Department of Agriculture’s Long-Term Agroecosystem Network, are one way to increase representation in documenting adaptation action led by underrepresented stewards, including people who identify as Indigenous, non-Anglo, female, or are recent rural migrants.^42^^,^^96^ Strategies such as stakeholder mapping, which involves identifying participant demographics, needs, and program participation, are another way to expand the representation of land steward perspectives in research, which can inform local policy, financial investment, and other adaptation planning.^8^^,^^42^^,^^97^ Quantitative indicators that can be measured to assess changes in stakeholder dynamics include shifts in occupation (e.g., % farmers, ranchers), age of landowners (e.g., mean age), or income level. Partnering with organizations already working to address geospatial, technical, and data sovereignty needs on Tribal lands, such as IndigiGenius (https://www.indigigenius.org/) or Tribal GIS (https://tribalgis.com/), can help ensure that adaptation strategies are informed by Indigenous knowledge and responsive to community priorities.

### Consider economic and environmental tradeoffs

Grassland management practices involve complex economic and environmental tradeoffs that shift across time and space.^98^ Federal, State, and Tribal grassland management agencies, nongovernmental organizations, and researchers should consider these tradeoffs when offering guidance to land stewards. Addressing woody plant encroachment, for example, often requires costly mechanical removal or prescribed burning, yet failure to do so reduces both local biodiversity and available forage, which may force cattle producers to reduce stocking rates and see a corresponding decline in profits.^99^ Regional efforts to defend rangeland cores from invasive grasses similarly impose collective costs, increasing financial strain to already resource-limited managers.^100^ As climate impacts intensify, these environmental and economic pressures are expected to cause additional financial challenges to the rangeland cattle industry and other key sectors.^101^

Grassland managers must account for the individual-level costs and benefits of climate-adaptive management practices, while also considering their intersection with broader social and systemic dynamics. Cost remains a primary barrier to adopting new grassland management strategies, particularly for small-scale producers who must continue to compete with well-resourced industrial operations while implementing new practices that require both time and money.^78^^,^^89^^,^^102^ Financial incentives that offset costs, such as stipends for conservation program participation or equipment, can encourage the adoption of new practices.^38^^,^^44^^,^^45^^,^^84^ Quantitative indicators that can be measured by conservation program managers include the proportion of local land stewards receiving financial incentives, as well as shifts in broader economic dynamics over time. In Kansas, farmers were more likely to plant bioenergy crops that mitigate climate change by reducing fossil fuel use if they had a secondary income to offset potential financial losses.^69^ In the Great Plains more broadly, agriculture census analyses from 1910 to 2017 show that long-term shifts in land ownership, production systems, and economics have progressively narrowed profit margins for farmers and increased exposure to market volatility.^16^ However, directing capital to support community ties and fostering a culture of stewardship and interdependency can encourage continued use of adaptation practices among land stewards.^61^ These findings underscore the importance of evaluating management practices not in isolation, but as part of a dynamic system of tradeoffs shaped by changing ecological conditions, economic pressures, and the practical realities land managers face over time.

### s-Frame strategies for supporting adaptation practices

Individual adoption of adaptation practices is enabled or constrained by system-level factors that are addressed with *s-frame* strategies for supporting adaptation practices. For example, while individuals may support native vegetation restoration to boost biodiversity and ecosystem resilience, economic or policy incentives favoring traditional crops or grazing can pose obstacles to implementing this adaptive strategy.^66^ Designing adaptation strategies that remove or lower systemic barriers can facilitate the adoption of adaptive management practices already supported by local individuals (e.g., ranchers) and social groups (e.g., grazing coalitions).^103^^,^^104^

We identified five *s-frame* solutions to support adaptation at the systemic level: 1) facilitate cooperative action between groups, 2) ensure “social fit” of grassland conservation practices and regulations, 3) identify overlapping benefits for social and ecological systems in adaptation planning, 4) assess the impacts of shifting grassland management to Great Plains communities, and 5) develop context-specific solutions.

### Facilitate cooperative action between groups

Cooperative action across stakeholder and rightsholder groups throughout the Great Plains is needed to conserve grasslands in a changing climate and facilitate learning across shifting social groups. Urbanization and amenity migration have disrupted once tight-knit communities that fear their way of life is threatened with the arrival of newcomers who may share different ecological values and perspectives on the best way to use and manage the land.^83^^,^^105^ Evolving regional ideologies, feelings of political underrepresentation, and lack of communication within and across stakeholder and rightsholder groups can exacerbate divisions between groups with opposing interests and ideas for management.^61^^,^^106^^,^^107^

Creating spaces to discuss differing motivations is essential for facilitating the adoption of climate adaptation practices.^83^ Grassland scientists may consider collaborative and participatory research to inform context-based solutions by involving community members from the project’s inception.^108^ Bringing together networks of diverse stakeholder groups can enhance producers’ consideration of more interconnected factors that shape decision-making about how to implement livestock production, conservation, carbon storage, and other ranching practices.^76^^,^^105^ Established and trusted rural networks that land stewards already operate within are conducive to sharing emerging information, tools, and practices related to evolving grassland management in a changing climate.^90^^,^^109^ Among Nebraska livestock producer networks, access to different sources of information within one’s own network predicted greater uptake of adaptive practices, such as prescribed burning.^110^ Networks organized by trusted groups, such as the World Wildlife Fund’s Ranch Systems and Viability Planning network, have also been successful in partnering with groups of ranchers to prevent cropland conversion through ranch-wide ecological monitoring, technical assistance, financial resources, and peer-to-peer learning.^111^ When convening network events, organizers should carefully consider format, as in-person engagement may be more accessible and effective than online delivery.^112^ Trusted experts who possess experience in both local and Western scientific contexts can serve to bridge the gap between different institutional scales in rangeland and natural resource contexts.^50^

Political leaders play a crucial role in facilitating cooperation and encouraging climate-resilient practices. Local and regional leaders can help bridge these divisions by setting social norms for adaptive action through communicating their policy endorsement to the public.^48^ Discussing the impacts of climate change on the local area can heighten its perceived severity and bolster support for local policy action, especially among Republican and Independent residents.^49^^,^^51^ Messaging from elected and community leaders can influence attitudes by promoting climate policies, with messages from in-group leaders shaping behavior more effectively than those from out-group leaders.^46^ Researchers can quantify the prevalence of climate-related messaging from political leaders by analyzing the frequency of relevant mentions in local news coverage or by conducting mixed-methods analyses of public comments, engagement with, or mentions of climate issues in social media content. Conservation professionals can engage these leaders in communication efforts that promote the use of climate-resilient grassland management practices.

### Ensure good “social fit” of grassland conservation policies and regulations

The success of conservation on working landscapes, where goals include supporting both human livelihoods and ecological health, heavily depends on “social fit,”^45^ which includes the social acceptance of conservation programs and their alignment with local well-being. This is particularly true in the Great Plains, where the majority of the land is privately owned and managed.^6^ By assessing relevant individual and social dimensions, policymakers, federal and state resource managers, and non-profit staff can identify opportunities for aligning conservation instruments with local needs and design systems that reduce the competition between conservation and socio-economic goals of stakeholders and rightsholders who manage the land. Working landscapes have shown significant promise in advancing conservation goals while also protecting human well-being.^113^^,^^114^ In the Canadian Prairies, for example, conservation policies have been designed to meet species-at-risk conservation goals, while also being compatible with agricultural operations, enabling sustained change.^52^

Policies that achieve good social fit by aligning with local preferences, values, interests, beliefs, and attitudes of local stakeholders and rightsholders tend to perform better.^27^ Enhancing the social fit of conservation policies depends on understanding contextually relevant needs of social and ecological environments, since motivators for adaptation often differ across federal, state, and Tribal governments, non-government organizations, and land stewards.^83^^,^^115^ For example, in the Southern Great Plains, where producers rely heavily on volunteer fire departments, neighbors, and partial insurance and disaster assistance following wildfire events, policies that invest in resources and support in those existing community networks can achieve a good social fit.^116^ Building understanding of social fit involves setting benchmarks for success, which may be achieved through listening sessions, public hearings, focus groups, surveys, or by partnering with community organizations to identify shared goals, build trust across polarized groups (e.g., political conservatives and liberals), and leverage local knowledge for conservation.^52^^,^^117^ Social fit can be measured quantitatively over time by establishing local benchmarks for success, then leveraging survey measures to track changes over time.

Conservation professionals at federal and state fish and wildlife agencies can help to inform good policies by highlighting their successes in using evidence-based management practices in collaboration with land stewards. While federal and state employees often do not write policies directly and are forbidden from lobbying, they can share with legislators and the public about the importance of grassland ecosystems and the work they are doing to conserve them in a changing climate. Environmental nongovernmental organizations can play a role in advocating for the enactment of specific policies and can help inform land stewards on policy changes and the opportunities or constraints they present for on-the-ground grassland management.^56^^,^^117^ Developing and revising policies for ongoing effectiveness requires communication and feedback among federal and state officials, Tribal Nations, the private sector, non-government organizations, municipalities, landowners, and the public.^118^

### Identify overlapping benefits for social and ecological systems in adaptation planning

Conservation professionals can more effectively partner with land stewards to implement and sustain adaptation practices by identifying strategies that offer benefits for both social and ecological systems. Targeting these “opportunity areas” can improve efficient use of limited resources (e.g., time, money, and personnel). Historically, socio-economic well-being and environmental conservation have often been pitted against each other,^119^ yet many of the papers reviewed for this synthesis^120^^,^^121^ highlighted the potential for adaptation practices that improve both ecological health and human well-being. For instance, removing invasive juniper trees from grassland ecosystems can reduce the rate of health issues caused by increased pollen production while also restoring native grass species and improving environmental health.^59^

When conservation professionals partner with land stewards to develop adaptation strategies that address the climate vulnerabilities of social and ecological systems, land stewards can more effectively meet their management goals within a changing climate, fostering resilience of grassland systems.^122^ Public policy can have a powerful effect on land cover and land use in the Great Plains, such as helping to drive the conversion of agricultural land back to grassland.^123^ In North Dakota, South Dakota, Nebraska, and Montana, policies that reduce subsidies and limit yield or revenue coverage have been passed to discourage sod tilling (Agricultural Act of 2014, P.L. 113-79). As water becomes increasingly scarce in the Great Plains, public policies that support drought-tolerant agricultural practices can help reduce demand on that resource and thereby increase water available for other important uses. For example, in eastern Kansas, regulating water use and offering incentives to grow drought-resistant crops can facilitate water conservation and improve resilience to drought.^69^ Well-designed policies can further enable ranchers to manage the land in a way that supports wildlife conservation while also bolstering the economic sustainability of ranches, rendering them more resilient to the negative impacts of climate change on biodiversity and water resource availability.^113^^,^^124^ Policy design should consider the proximity of benefits, since adaptation practices with future returns can experience lower adoption than those offering immediate gains. Ecological indicators such as land use patterns, drought risk, and the conservation status of wildlife can be combined to identify priority areas for adaptation efforts.

### Assess the impacts of shifting grassland management to Great Plains communities

Assessing how shifting land ownership patterns interact with changing land use and social dynamics over time is important for designing strategies to encourage the adoption of climate-adaptive grassland management practices in the Great Plains. Rural communities across the Great Plains are experiencing demographic and land use changes brought about by a combination of successional challenges for ranchers^61^ and amenity migration^60^ (i.e., the movement of people to an area for the available environmental amenities). This transition from traditional production-oriented land use to recreational enjoyment of natural resources reshapes long-held communal values about grasslands and their management, posing challenges for environmental conservation.^83^^,^^125^ Ranching communities often have conservative values and may be less likely to prioritize environmental conservation for its own sake, while newcomers may value environmental protection more highly^63^; however, newcomers may be less effective at implementing conservation practices due to a lack of familiarity with the local ecosystem and available conservation programs.^34^^,^^64^^,^^66^ The generational, experiential knowledge that many ranchers possess is likely crucial to the successful conservation of grasslands in a changing climate. This issue is exacerbated by absentee landlords in areas experiencing amenity migration, as some may reside outside the region and are not present to detect and address problems as they arise.^83^

Successional challenges and amenity migration directly contribute to the fragmentation of the grassland landscape. Many ranches sold to outsiders are not sold in one piece, but rather subdivided and sold to those wishing to live on a “ranchette.” As the local population and affluence increase, the grassland ecosystems may be further fragmented by the development of new roads, sewers, schools, and recreational infrastructure (e.g., airports, golf courses, and ski resorts).^84^ Quantitative indicators such as rates of parcel subdivision, changes in housing density, road-network expansion, and shifts in local demographic or income patterns can help measure how these dynamics contribute to grassland fragmentation. An *s-frame* adaptation opportunity for researchers, state and federal resource managers, and non-government organization staff is to assess how these shifting social dynamics influence local adaptation needs to inform relevant strategies for supporting resilient grassland management.

### Develop context-specific solutions

Developing context-specific solutions to social and environmental challenges posed by climate change can offer land stewards a set of adaptation practices to choose from that already account for the shifting dynamics they are navigating. Conservation professionals involved in adaptation planning—such as federal and state resource managers, nonprofit-organization staff, and Tribal leaders—can support land stewards with resilient grassland management at a broader scale by leveraging understanding of the social contexts to inform which adaptation practices in their region to recommend, incentivize, and design policy around. This requires identifying social dimensions at both individual and systemic levels for a broad array of land stewards, such as existing knowledge, barriers to implementing resilient practices, and locally experienced environmental challenges.^68^^,^^122^^,^^126^

Mapping social and economic factors geospatially (i.e., public participatory geographic information system mapping-based research [PPGIS]) is an underutilized tool that could help to provide insights on grassland management needs and changing social and ecological dynamics, which can then be used to inform adaptation from small to large scales.^66^ PPGIS can be used to identify relevant demographic dimensions in the area, design communications for target audiences, and inform context-specific solutions that incorporate management needs of a broad array of stakeholders and rightsholders. Such techniques have been successfully employed by government research agencies in other regions^96^ and offer a framework for collecting localized data to inform context-specific management solutions. PPGIS is a method that interacts with both system-level (*s-frame*) and individual-level (*i-frame*), in that it collects quantitative data at the individual level to inform adaptation, informing education and outreach efforts to land stewards, while also providing broader insights at the systemic level about which policies, resources, and forms of adaptation are most needed in a particular region.

## Discussion

Through our synthesis of the social science literature, we have identified nine social-science-backed strategies—four focused on individual-level (i.e., *i-frame*) interventions and five focused on systemic-level (i.e., *s-frame*) interventions—that conservation professionals can implement to support adaptation to climate change in the Great Plains. However, as others have highlighted,^127^^,^^128^^,^^129^^,^^130^ the *i-frame* and *s-frame* categories may be overly simplistic. In this discussion, we explore both the value of focusing on *i-frame* and *s-frame* strategies for implementation by conservation professionals and opportunities for developing a more nuanced framework. Specifically, we discuss the utility of the *i-frame* and *s-frame* for supporting conservation professionals in promoting climate adaptation in the Great Plains and in grasslands around the world. We then discuss the limitations of the *i-frame* and *s-frame* categories and the opportunity for future research and synthesis to more fully explore an additional, group-level frame for intervention. Finally, we highlight underrepresented groups in our synthesis.

### Applying i-frame and s-frame strategies in the Great Plains and beyond

In the Great Plains, conservation professionals may work for federal, state, local, or Tribal agencies or non-government agencies. Regardless of their employer, these professionals often work toward their goals by helping individual landowners implement conservation practices, or setting institutional or regional policies and plans to support conservation. By using the *i-frame* and *s-frame* categories developed by behavioral economists, we were able to highlight strategies that target intervention levels most relevant to the work of these conservation professionals. Conservation professionals who routinely work with individual landowners could apply the *i-frame* strategies to support their clients in adapting to climate change, while those conservation professionals who work at the level of strategic planning and policymaking could apply the *s-frame* strategies to enable adaptation at a larger scale.

Importantly, both *i-frame* and *s-frame* strategies are needed to enable timely and effective adaptation. When conservation professionals focus exclusively on *i-frame* strategies, stakeholders and rightsholders are left to bear the full burden of response, which can pose barriers to action and limit the scalability of adaptation practices.^70^^,^^121^ Conversely, solely focusing on *s-frame* strategies to change the systems within which individual decision-makers operate may fall short if the values, concerns, motivations, and ideologies that shape individual decisions.^11^ By supporting adaptation to climate change through a combination of both *i-frame* and *s-frame* strategies, conservation professionals can lower barriers for land stewards to make decisions that can benefit both the ecological and social systems in grassland regions.

Though the nine *i-frame* and *s-frame* strategies presented here were synthesized from a review of social science literature focused on the Great Plains, these strategies are nevertheless relevant for supporting adaptation in grasslands around the world. Grassland conservation and restoration have been legally mandated in a number of countries^131^ and best practices for restoring grasslands have been identified^131^; however, to our knowledge, there has not been a similar effort to identify strategies or best practices for addressing the human dimensions of grassland conservation, which are equally important to the success of those efforts. The nine *i-frame* and *s-frame* strategies presented here can be applied to address the human dimensions of grassland management and conservation around the world and represent a first step at filling this knowledge gap. Furthermore, these general strategies could also be applied to supporting adaptation to climate change in other ecosystems.

### Developing group-level strategies to support adaptation in grasslands

While this review and synthesis of the Great Plains social science literature focused on identifying *i-frame* and *s-frame* strategies to align with conservation professionals’ typical scope of work, these categories are neither entirely discrete nor comprehensive. Similar to Chater and Loewenstein^11^ we found that the boundary between *i-frame* and *s-frame* was not always clear. For example, facilitating cooperation between groups requires intervention at the individual level (e.g., making individuals aware of common goals) and, in many cases, interventions at the systemic level (e.g., creating safe spaces for diverse groups to gather). However, the current conceptualization of the *i-frame* and *s-frame* framework does not define a clear set of criteria for categorizing strategies that operate both within the *i-frame* and *s-frame*, nor group-level interactions.

Furthermore, as Bingley and colleagues argue,^128^ focusing only on the individual and systemic levels overlooks the crucial middle level that links the two: the group level, or *g-frame*. They call for a *g-frame* analysis that “focuses on how cognition and behavior are shaped and transformed by membership in social groups.” While a comprehensive definition of the *g-frame* remains to be formally developed since Chater and Loewenstein’s original framework paper,^11^ a *g-frame* analysis may include how individual thinking and actions are influenced by group and social dimensions including collective norms, values, and beliefs^132^; shared knowledge and experiences^133^; common goals^70^; rural identity and culture^84^; and intergroup and intragroup relationships.^46^ Therefore, *g-frame* interventions are grassroots interventions that focus on enabling collective action in which individuals are empowered to enact change through their membership in social groups.^128^ For example, in grassland areas experiencing amenity migration, long-term ranchers and newer residents’ grassland management decisions may be influenced as much by their desire to fit in with the group they identify with (e.g., long-time ranchers or hobby farmers) as by their individual capacity to enact certain practices or the structural constraints and opportunities available to them. As Bingley and colleagues argue, collective action at the group level could undoubtedly be powerful for supporting adaptation to climate change in grasslands; however, unless conservation professionals are already members of a relevant group, they may find it more difficult to initiate these actions. Yet, by employing the *i-*frame and *s-frame* strategies described above, conservation professionals can help to support conditions that are favorable to the emergence and success of collective grassland adaptation action at the group or grassroots level.^133^ Further development of the relevance and application of the *g-frame* to behavior interventions related to climate adaptation is a ripe area for future research and synthesis.

### Underrepresented groups in social science literature

Similar to Bruno and colleagues,^8^ we found that the social science literature did not evenly represent all stakeholder and rightsholder groups in the Great Plains. In the literature reviewed for this paper, ranchers, landowners, and farmers were most often the focus, while there were comparatively few articles and reports focused on Indigenous peoples or Tribal Nations and corporations. Future reviews may address this gap by extending to regions beyond the Great Plains to capture the wider and rapidly growing literature on land stewardship and may include targeted search terms such as “Traditional Ecological Knowledge,” “land stewardship,” and “Indigenous knowledge.” The strategies we present in this paper may therefore be more appropriate for supporting climate adaptation among ranchers, landowners, and farmers than Indigenous peoples, Tribal Nations, and corporations. Further research is needed to better understand the climate adaptation goals, capacities, and needs of Indigenous peoples, Tribal Nations, and corporations in the Great Plains, and to what extent lessons from more well-represented groups are applicable.

### Quantitative indicators

Monitoring and evaluating the effectiveness of adaptation—and strategies to promote uptake of adaptation approaches—is an area of growing importance.^134^^,^^135^^,^^136^^,^^137^ Recent work to identify quantitative metrics to evaluate adaptation has focused on measuring ecological and biological outcomes,^137^^,^^138^^,^^139^^,^^140^^,^^141^^,^^142^ while work on social processes or outcomes has focused more on qualitative indicators.^136^^,^^143^^,^^144^ Research on qualitative indicators of adaptation success from the social sciences can therefore be an important precursor to developing robust quantitative metrics to support monitoring and evaluation efforts, and is a growing area of scholarship that merits additional future research. To track the impact and effectiveness of the nine social science strategies we have presented, conservation professionals may measure quantitative indicators over time. While a comprehensive review of quantitative metrics for adaptive grassland management is outside of the scope of this social science review, we present options for measurement tools relevant to each strategy and cite examples of relevant articles to facilitate further investigation (Table 2).

**Table 2:** Quantitative indicators that can be used to measure outcomes related to the nine social science strategies we present in this review

Strategy	Examples of Quantitative Indicators	Relevant Metrics
***i-frame***		

Tailor education and communication	changes in risk perception adaptation attitudes	risk perception (*Likert scale of agreement*)^73^ attitudes toward the importance of adaptation to the long-term success of U.S. agriculture (*Likert scale of importance*)^22^
Explain the implications of policy changes in educational outreach	public awareness of policies changes in understanding over time	awareness of laws/regulations for prescribed burn bans (*Likert scale of agreement; self-report*)^93^ change over time in the proportion of residents who understand the purpose of prescribed burning (*0 =no, 1 =yes, 2 =don’t know)*^145^
Include diverse groups and individuals in research and engagement	age of residents gender	age range, mean, and median^146^ sex by occupation^146^
Consider economic and environmental trade-offs	program involvement or financial support changes in economic dynamics over time	counts of participation in the EQIP program (*0 =no, 1 =yes*) or amount of funding (*$ USD; self-report)* awarded through the EQIP Conservation Innovation Grant^23^ percentage of farmer income from agriculture (*%; self-report*)^43^

***s-frame***		

Facilitate cooperation between groups	membership in existing networks	rates (%) of landowner membership in prescribed burn association groups (0 = no, 1 = yes)^147^
Ensure “social fit” of grassland conservation practices and regulations	presence of existing policies public attitudes toward policies	state has a climate action plan (*0 =no, 1 =yes*)^49^ support for specific policies (*-3 =strongly oppose to +3 =strongly support; self-report*)^148^
Identify overlapping benefits for social and ecological systems in adaptation planning	ecological: drought risk economic: industries that support the local economy	drought intensity classification scores (*D0 =abnormally dry to D4 =exceptional drought)* in a defined area^149^ gross domestic product by industry and state^150^
Assess the impacts of shifting grassland management on Great Plains communities	changes in housing density shifts in local demographics	counts or rates (%) of housing units in a given area over time^146^ changes in age, ethnicity, or household composition in a defined location over time^146^
Develop context-specific solutions	community priorities for conservation perceived local barriers to adaptation	ranking priorities from a list of local issues; attitudes toward grazing/other practices (*Ranking; Likert scale of agreement*)^151^ barriers related to personal belief, farm characteristics, economics, or education among farmers (*Likert scale of agreement*)^43^

## Conclusion

Grassland management decisions are important for the resilience of grassland ecosystems in a changing climate; these decisions are affected by both individual and systemic dimensions, as well as their interaction.^37^^,^^38^^,^^45^^,^^48^^,^^152^ However, in the U.S. Great Plains, adaptation efforts by conservation professionals have largely targeted individual land stewards, emphasizing technical assistance and practice adoption at the ranch or farm level, while less focus has been placed on transforming the broader systems that influence land-use decisions and long-term resilience.^33^^,^^153^ Scaling climate adaptation across the region will require both systemic and individual changes to make the implementation of new management practices viable for land stewards.

This synthesis offers conservation professionals nine social-science-backed strategies for supporting land stewards to adopt climate adaptation efforts in grassland ecosystems. The *i-frame* and *s-frame* provide useful categorization tools to identify adaptation strategies for grassland management because the framework identifies methods to support both land stewards individually, as well as changing the broader systems in which decision-makers operate. Our findings also underscore the need for a more nuanced framework that includes social or group-level (*g-frame*) dynamics. Future research should explore how *g-frame* interventions can empower collective action and better reflect the social realities of conservation work. Addressing this gap will be essential for ensuring long-term, equitable, and effective adaptation strategies in grassland conservation and management.

### Limitations of the study

This synthesis has reviewed the social sciences relevant to climate change adaptation in the Great Plains. Our review is not without limitations. First, while we present a novel application of the *i-frame* and *s-frame* framework from behavioral economics to a grassland management context, it is outside of the scope of our review to formally develop a third branch of the framework focused on group dynamics: the *g-frame*. Second, while we provide examples of quantitative indicators that can be used to track the success of the social science strategies we present, this review is not intended to summarize measurement approaches in-depth, nor does it include qualitative indicators, which can be informative in understanding social change over time. Lastly, given that all stakeholder and rightsholder groups in the Great Plains were not represented evenly, we are careful not to extend our findings to groups that were less represented in our review, such as Indigenous peoples, Tribal Nations, and corporations.

## Acknowledgments

The authors wish to thank Sarah Jaffe for preparing Figure 1 for this manuscript and Creigh Rourke for supporting the literature review. This material is based on work completed as part of the North Central Climate Adaptation Science Center Rapid Climate Assessment Program, which was supported by the U.S. Geological Survey North Central Climate Adaptation Science Center under Cooperative Agreement No. G18AC00325*.* The views and conclusions contained in this document are those of the authors and should not be interpreted as representing the opinions or policies of the U.S. Geological Survey. Mention of trade names or commercial products does not constitute their endorsement by the North Central Climate Adaptation Science Center or the U.S. Geological Survey.

## Author contributions

Author contributions based on the Creditor Roles Taxonomy (CRediT). Conceptualization and methodology (S.G.C., C.D.M.H, H.M.Y., and R.R.) investigation and data curation (S.G.C.), writing – original draft (S.G.C., C.D.M.H, H.M.Y., and R.R.), writing – review and editing (S.G.C., C.D.M.H, H.M.Y., and R.R.), visualization (S.G.C, C.D.M.H., and H.M.Y.), supervision (C.D.M.H. and H.M.Y.), project administration (S.G.C, C.D.M.H., and H.M.Y.), funding acquisition (C.D.M.H. and H.M.Y.).

## Declaration of interests

The authors declare no competing interests.
