# Supplementary material for: Social science for climate-resilient grasslands: Strategies to support individual and systemic adaptation
Source: iScience. 2026 Jun 8;29(6):116265. doi: 10.1016/j.isci.2026.116265 (PMC13264105; doi:10.1016/j.isci.2026.116265)
Supplement: Document S1. Tables S1–S3 [file mmc1.pdf]

iScience, Volume *29*

Supplemental information

Social science for climate-resilient grasslands:

Strategies to support individual

and systemic adaptation

Sarah Gonzalez-Coffin, Christine D. Miller Hesed, Heather M. Yocum, and Ryan Roberts

Table S1.
Glossary of terms referenced in the manuscript.

Term	Definition
Adaptation	Adjustments made in response to actual or expected climatic stimuli that aims to reduce vulnerability to climate change impacts
Anthropology	The study of human societies and cultures and their development
Communication	An academic discipline that deals with processes of human communication and behavior, patterns of communication in interpersonal relationships, and social interactions
Conservation Professionals	Those who design, fund, and implement natural resource management plans in collaboration with land stewards
Corporation	Specific legal form of organization of persons and material resources for the purpose of conducting business
Economics	The branch of knowledge concerned with the production, consumption, and transfer of goods and services
Education	An academic discipline concerned with methods of teaching and learning in schools or school-like environments
Ejido	An area of communal land used for agriculture in which community members have usufruct rights rather than ownership rights to land
i-frame	A structured conceptual model for framing problems and solutions in terms of individual behaviors
Individual Dimensions	Personal characteristics that may affect the decisions about grassland management
Indigenous	Used to refer to people who are the original occupants in a place
Land Steward	An owner, proprietor, or caretaker of a piece of land including private landowners
Mitigation	Refers to actions aimed at reducing or preventing the emission of greenhouse gases that contribute to climate change
Natural Science	An academic discipline and branch of science that deals with the physical world and lifeforms on it
Non-Governmental Organization	An organization that functions independently of any government
Pastoralist	A farmer who breeds or takes care of animals
Political Science	An academic discipline and branch of knowledge that deals with systems of government, specifically the analysis of political activity and behavior
Physical Science	An academic discipline and the sciences concerned with the study of inanimate or inorganic natural objects
Psychology	A scientific discipline that studies mental states and processes and behavior in humans and other animals
Rancher	A person who owns or works on a ranch, a large farm, or estate to raise livestock

Rightsholder	A person who holds specific treaty rights
s-frame	A structured conceptual model for framing problems and solutions in terms of social systems
Social Science	A branch of science that focuses on the study of people as individuals, communities, and societies
Sociology	An academic discipline that studies of the development, structure, and functioning of human society
Stakeholder	An individual or a group of individuals with an interest in the success of something
Systemic Dimensions	The overarching systems, regulations and resource availability that stakeholders and rightsholders operate within

Table S2. Social Science Information Needs Identified by the Complementary Ecological Synthesis

The relationships between the individual level and systemic dimensions, as well as related i-frame and s-frame approaches for supporting adaptation practices, and the fifteen broadly shared information needs identified by the complimentary ecological synthesis [S1, S2]. A symbol of "X" indicates the relevance of a key finding discussed in the paper to the broadly shared information needs.

Broadly Shared Information Needs															
1. Where are grasslands most likely to be lost to other land uses?<br>2. What are best practices for grassland restoration in a changing climate?<br>3. How will climate change affect disturbance regimes?<br>4. How will climate change impact woody encroachment?<br>5. How will climate change impact herbaceous invasives?<br>6. How will climate change impact grazing?<br>7. How will climate change impact water quality, quantity, and availability?<br>8. How will climate change affect animal species of conservation concern?<br>9. How can conservation on private grasslands be achieved?<br>10. How can public understanding of grasslands and their importance increase?<br>11. What legal and policy changes can support grassland resilience to climate change?<br>12. How can grassland protection, enhancement, maintenance, and reconstruction be economically incentivized?<br>13. How can grassland management be strategically coordinated across agencies, organizations, jurisdictions, and borders?<br>14. How can the accessibility of relevant science and tools be improved?<br>15. What novel ways of thinking are needed to successfully manage grasslands amidst climate change?															
Key Findings	Broadly Shared Information Needs														
	1	2	3	4	5	6	7	8	9	10	11	12	13	14	15
Individual Level Human Dimensions															

Individual Dimensions Relevant to Grassland Management															
Differences in Attitudes, Values, and Ideologies	X	X	X	X	X	X	X	X	X						X
Differences in Risk Perception	X	X	X	X	X	X	X	X	X	X					X
Demographic Variation	X	X							X						
Intergroup Cooperation and Understanding	X	X					X				X		X	X	X
i-Frame Approaches for Supporting Adaptation Practices															
Tailor Education and Communication			X	X				X		X				X	X
Ensure Diverse Groups and Individuals Included in Research and Engagement	X											X		X	X
Consider Economic and Environmental Tradeoffs	X	X	X							X		X	X		
Facilitate Cooperative Action					X						X		X	X	X
Systemic Level															
Systemic Dimensions Relevant to Grassland Management															
Demographic and Land Use Changes	X	X	X										X		

Interdependencies Between Individuals, Societies, and Systems			X	X							X				
Existing Practices and Economic Pressures	X	X		X		X	X	X	X						
Policies and Regulations				X							X				
s-Frame Approaches for Supporting Adaptation Practices															
Ensure Good “Social Fit” of Grassland Conservation Policies and Regulations	X	X					X	X	X		X	X	X		X
Explain the Implications of Policy Changes in Educational Outreach											X	X	X	X	X
Identify Overlap Between Social and Ecological Systems in Adaptation Planning	X	X	X	X	X	X	X	X	X				X		
Assess the Impacts of Shifting Grassland Management to Great Plains Communities	X	X					X		X						
Develop Context-Specific Solutions	X	X	X	X	X	X	X	X	X	X	X	X	X		X

Table S3. Number of Studies Relevant to Each Information Need

Frequency of articles (n = 203) that addressed the 15 broadly shared information needs to support successful grassland management in a changing climate, based on prior syntheses [S1, S2]. Articles were counted as having addressed a question if they presented one or more answers to the question posed. Note that most studies answered more than one question; frequencies are not discrete.

Social science information need, identified in the ecological synthesis	Number of studies in the social synthesis that addressed this question
How can conservation on private grasslands be achieved?	78
What are best practices for grassland restoration in a changing climate?	68
What novel ways of thinking are needed to successfully manage grasslands amidst climate change?	56
Where are grasslands most likely to be lost to other land uses?	50
How can grassland protection, enhancement, maintenance, and reconstruction be economically incentivized?	43
How will climate change affect disturbance regimes?	26
How can public understanding of grasslands and their importance increase?	23
How can grassland management be strategically coordinated across agencies, organizations, jurisdictions, and borders?	21
What legal and policy changes can support grassland resilience to climate change?	21
How can the accessibility of relevant science and tools be improved?	19
How will climate change impact water quality, quantity, and availability?	18
How will climate change impact woody encroachment?	15
How will climate change impact grazing?	11
How will climate change affect animal species of conservation concern?	7
How will climate change impact herbaceous invasives?	6

Supplemental References

[S1] Miller Hesed, C. D., & Yocum, H. M. (2023). Grassland management priorities for the North Central Region. In *Open-File Report* (2023–1037). U.S. Geological Survey. https://doi.org/10.3133/ofr20231037

[S2] Miller Hesed, C. D., Yocum, H. M., Cross, M., Bamzai-Dodson, A., Wheeler, B., Beckmann, J. P., Ahlering, M., Hall, K. R., Boyd-Valandra, E., Mosher, D., Miller, B. W., & Jaffe, S. (2023). Seventy questions of importance to the conservation of the North Central grasslands of the United States in a changing climate. *Conservation Science and Practice*, e12998. https://doi.org/10.1111/csp2.12998
